# Temporal Variation of Chlorophyll-a Concentrations in Highly Dynamic Waters from Unattended Sensors and Remote Sensing Observations

**DOI:** 10.3390/s18082699

**Published:** 2018-08-16

**Authors:** Jian Li, Liqiao Tian, Qingjun Song, Zhaohua Sun, Hongjing Yu, Qianguo Xing

**Affiliations:** 1 School of Remote Sensing and Information Engineering, Wuhan University, Wuhan 430079, China; lijian@whu.edu.cn; 2 State Key Laboratory of Information Engineering in Surveying, Mapping and Remote Sensing, Wuhan University, Wuhan 430079, China; tianliqiao@whu.edu.cn; 3 National Satellite Ocean Application Service, State Oceanic Administration, Beijing 100081, China; 4 Key Laboratory of Space Ocean Remote Sensing and Application, State Oceanic Administration, Beijing 10081, China; 5 State Key Laboratory of Tropical Oceanography, South China Sea Institute of Oceanology, Chinese Academy of Sciences, Guangzhou 510301, China; Joeysun@scsio.ac.cn; 6 State Key Laboratory of Simulation and Regulation of Water Cycle in River Basin, Institute of Water Resources and Hydropower Research, Beijing 100038, China; yuhj@iwhr.com; 7 Key Laboratory of Coastal Environmental Processes and Ecological Remediation, Yantai Institute of Coastal Zone Research, Chinese Academy of Sciences, Yantai 264003, China; qgxing@yic.ac.cn

**Keywords:** chlorophyll-a, remote sensing, Poyang Lake, temporal scale, sampling strategy

## Abstract

Monitoring of water quality changes in highly dynamic inland lakes is frequently impeded by insufficient spatial and temporal coverage, for both field surveys and remote sensing methods. To track short-term variations of chlorophyll fluorescence and chlorophyll-a concentrations in Poyang Lake, the largest freshwater lake in China, high-frequency, in-situ, measurements were collected from two fixed stations. The K-mean clustering method was also applied to identify clusters with similar spatio-temporal variations, using remote sensing Chl-a data products from the MERIS satellite, taken from 2003 to 2012. Four lake area classes were obtained with distinct spatio-temporal patterns, two of which were selected for in situ measurement. Distinct daily periodic variations were observed, with peaks at approximately 3:00 PM and troughs at night or early morning. Short-term variations of chlorophyll fluorescence and Chl-a levels were revealed, with a maximum intra-diurnal ratio of 5.1 and inter-diurnal ratio of 7.4, respectively. Using geostatistical analysis, the temporal range of chlorophyll fluorescence and corresponding Chl-a variations was determined to be 9.6 h, which indicates that there is a temporal discrepancy between Chl-a variations and the sampling frequency of current satellite missions. An analysis of the optimal sampling strategies demonstrated that the influence of the sampling time on the mean Chl-a concentrations observed was higher than 25%, and the uncertainty of any single Terra/MODIS or Aqua/MODIS observation was approximately 15%. Therefore, sampling twice a day is essential to resolve Chl-a variations with a bias level of 10% or less. The results highlight short-term variations of critical water quality parameters in freshwater, and they help identify specific design requirements for geostationary earth observation missions, so that they can better address the challenges of monitoring complex coastal and inland environments around the world.

## 1. Introduction

Inland lakes represent approximately 87% of the Earth’s surface freshwater. They provide important habitats and function within a variety of ecosystems [1], for example, they play a vital role in global carbon and nutrient cycles [2]. The water quality of inland lakes is, however, affected by multiple factors, including human population growth, industrial expansion, agriculture, and climate change [3,4,5]. The impacts of declining water quality on ecosystems, as well as on the normal lives of human beings, has been well recognized [6,7]. There is, therefore, an urgent need for effective monitoring and regulation of water resources, and a better understanding of the relationships between diurnal and annual solar variations, biological growth, and ecological processes.

For decades, field measurements of water quality changes have mainly depended upon in situ sampling and land-based or shipboard laboratory analysis. This yields highly precise results, but is costly, time-consuming, and labor-intensive [8]. Traditional field sampling methods are also often limited in terms of spatial and temporal coverage and resolution making it difficult to derive statistically meaningful results [9,10]. In this respect, monitoring of water resources by remote sensing is superior, as it has wider spatial coverage, long-term data acquisition stability, and lower costs when compared to conventional methods [11,12]. A great deal of effort has been invested into developing methods for monitoring water quality at global, regional, and local scales using remote sensing technology. This has resulted in the rapid development of systems referred to as ocean color radiometry satellites [13]. Conventional ocean color sensors included the proof-of-concept Coastal Zone Color Scanner (CZCS, 1978–1986), while more modern radiometers include the Sea-viewing Wide Field-of-view Sensor (SeaWiFS, 1997–2010), the Moderate Resolution Imaging Spectroradiometer (Terra/Aqua MODIS, 1999–present), and the Medium Resolution Imaging Spectrometer (MERIS, 2002–2012). Theoretically, these polar orbit satellite sensors provide comprehensive information about water properties at varied spatial and temporal scales [14,15]. Unfortunately, the ability of existing sensors to monitor highly dynamic bodies of water is insufficient, especially for regions with significant diurnal or semi-diurnal variations, and where satellite observations are frequently impacted by extensive cloud coverage and other phenomena, such as solar reflections [16,17,18].

For highly dynamic coastal and inland waters, significant short-term variations have frequently been observed in water quality parameters, such as turbidity, chlorophyll fluorescence, chlorophyll (Chl-a) levels, and colored dissolved organic matter (CDOM) levels [16,19]. High resolution spatio-temporal data are required, for example, to track diurnal changes of harmful algal blooms in the East China Sea, Tampa Bay, and Taihu Lake [20,21]. Significant diurnal changes of turbidity in coastal/inland waters implied that hourly observations were necessary, and it is necessary to be cautious when field sampling and remote sensing data are used to map turbidity or light attenuation in coastal waters, such as the southern North Sea and Hangzhou Bay [22,23]. With increasing demands for high-frequency monitoring of water quality, geostationary-orbit (GEO) ocean color satellites have become more common. South Korea launched the first GEO ocean color satellite, the Geostationary Ocean Color Imager (GOCI) in 2010 [16], ESA launched the Spinning Enhanced Visible and Infrared Imager (SEVIRI) in 2012 [24], and China’s first GEO satellite (GF-4) was launched in 2016. In addition, the Geostationary Coastal and Air Pollution Events (GEO-CAPE) system is under construction at NASA [25]. Although these GEO satellites are expected to provide high-frequency observations of water quality in dynamic coastal/inland regions, polar orbit satellites are still the standard in water quality remote sensing monitoring [13].

In situ chlorophyll-a (Chl-a) fluorescence is a proxy for Chl-a concentration, and hence phytoplankton biomass [26,27]. Datasets representing solar-stimulated chlorophyll fluorescence are available from satellite sensors, such as MERIS and MODIS, and they could be much more widely used for monitoring coastal bio-product yields and plankton blooms [28]. Consequently, there is an urgent need to better understand short-term variability in biological, optical, and chemical properties of coastal/inland waters, to determine temporal variations, and identify optimum sampling strategies of water quality, especially for Chl-a.

A fundamental concern is to identify the factors that affect the optical properties of water [29]. Furthermore, this will provide the foundations for further investigations of short-term variability in water quality [30]. With respect to the measurement systems, the ongoing satellite ocean color missions should allow a more thorough consideration of the trade-offs between water resource monitoring requirements and instrument design factors and limitations to take place.

To help advance this research and develop this methodology, this paper provides first-hand results regarding short-term variations and temporal scales for the key water quality parameter Chl-a, based on time series remote sensing data, and high frequency fixed-station field measurements taken at Poyang Lake, the largest freshwater lake in China. The sampling biases for Chl-a levels derived from Terra/Aqua MODIS observations were then estimated, the optimal sampling strategy was determined, and recommendations for water quality monitoring, both for routine field survey and high-frequency satellite missions, are made.

## 2. Study Area

### 2.1. Environment of Poyang Lake

**Figure 1:**
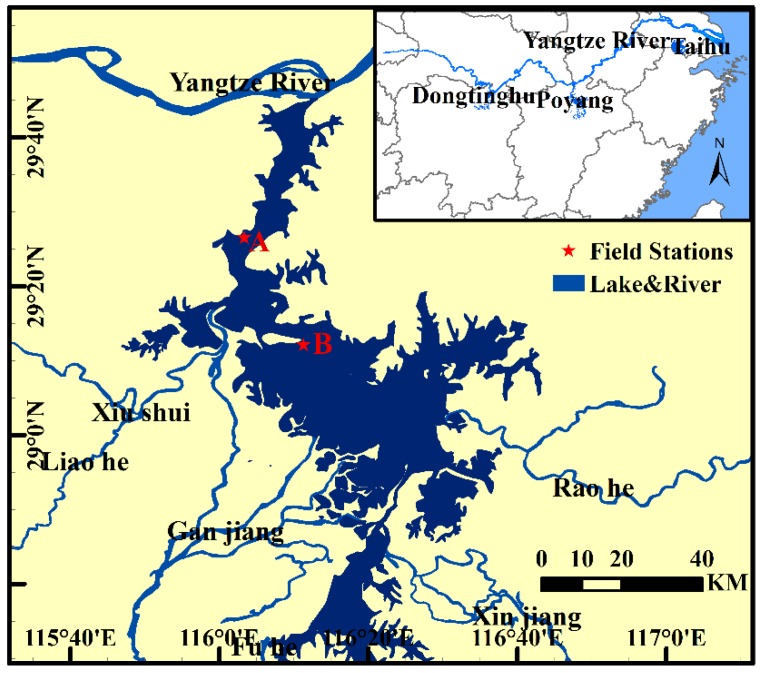
Location of Poyang Lake. The lake has five main tributaries and connects to the Yangtze River at Hukou. Red stars represent the field stations

Poyang Lake is located in the mid to lower Yangtze River valley in China (28°22′–29°45′ N and 115°47′–116°45′ E, Figure 1). It is the largest freshwater lake in China and the water area varies from approximately 3000 km^2^ during wet seasons to 1000 km^2^ during dry seasons, and is dependent on subtropical monsoons [31]. Poyang Lake and its associated wetlands provide one of the largest bird conservation areas in the world, supporting millions of birds and over 300 different species [32]. The ecological condition of Poyang Lake, however, is degrading due to more frequent droughts [33], eutrophication [34], and increasing pressure from human activities such as sand dredging [35].

Existing studies have primarily focused on water quality monitoring using MODIS [36], Landsat TM/ETM+ [37], or HJ-1 CCD [38] data at various spatial and temporal scales. The long-term (inter-annual and seasonal) dynamics of the lake, such as increasing turbidity and declining water quality, have been well documented [36,39,40]. The potential risk from cyanobacteria blooms was also evaluated [41,42,43], but there is a lack of information regarding the short-term variations of Chl-a, which is a factor of critical importance when monitoring aquatic environments.

### 2.2. In Situ Unattended Sensors and High Frequency Data Measurements

**Figure 2:**
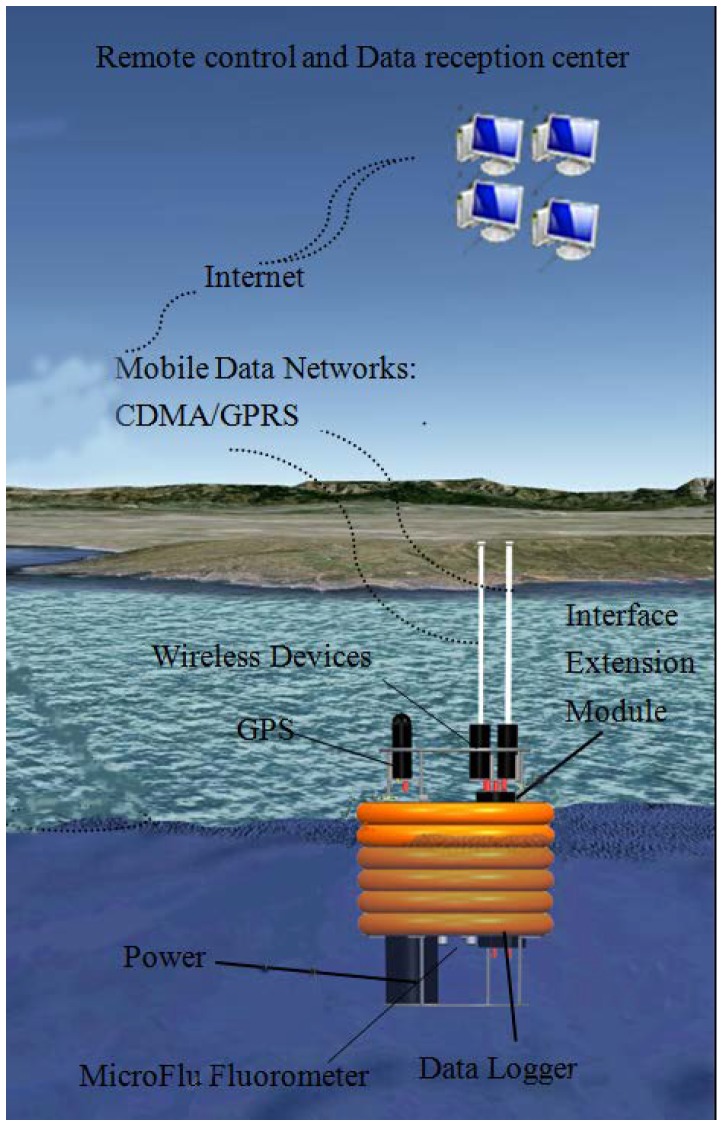
The schematic diagram of the in situ unattended sensor system and platform.

A suite of autonomous bio-optical and meteorological sensors was deployed at Poyang Lake to conduct high frequency measurements of water quality parameters, together with simultaneous measurement of local climatic conditions (wind speed, solar radiance), as showed in Figure 2. The WETLabs Environmental Characterization Optics ECO-FL sensors used were designed to measure chlorophyll-a using a series of fluorimeters. The sensors provide unattended continuous measurements using internal memory and batteries. All of the sensors were mounted in buoys and deployed at a bottom depth of 2.5 m. Instrument-specific calibration was carried out using WETLabs’ supplied scale factors, dark counts, and other characterization values. The HOBO weather suite (Onset Corporation, Bourne, USA), was installed in combination with plug-and-play smart sensors to measure air temperature, relative humidity, air pressure, solar radiance, wind speed, and wind direction. The observation period for site A ran from 20 to 28 July 2013, and for site B from 17 to 20 September 2014. The sampling interval was set at 30 s for all of the sensors. The validation of the ECO-FL measurements collected was conducted by a laboratory analysis, which is discussed in Section 4.2.

The in situ chlorophyll-a concentration data was collected using a lab-based fluorometric analysis method. The chlorophyll samples were first collected by being filtered through 0.45 μm Whatman cellulose acetate membranes. Back in the lab, the filters were soaked with ethanol (90%) at 0 °C for 24 h for extraction. The concentration of chlorophyll (Chl) (μg/L) was then determined by measuring the extracted pigment samples using an RF-5301 Fluorescent Spectrophotometer (Shimadzu, Kyoto, Japan), which was calibrated to the Chl-a standards determined by the Sigma Chemical Co. (St. Louis, MO, USA) before the main experiment was carried out. The exciting wavelength and fluorescent wavelength were 432 and 667 nm, respectively [44].

## 3. Materials and Methods

### 3.1. Spatio-Temporal Clustering of Time-Series Remote Sensing Data

The representativeness of the field station locations is crucial to obtaining reliable in situ data for monitoring water quality, especially for regions where high spatial and temporal variabilities were observed. In this study, the most representative station positions were determined using the spatio-temporal clustering method on long-term remote sensing images. Level-1 MERIS data (reprocessed versions of R2012.1) covering the study region were obtained from NASA’s Goddard Space Flight Center (GSFC). In total, more than 700 full resolution data “granules” (i.e., individual scenes) from the entire satellite mission, which ran from January 2003 to April 2012, were obtained. After visual examination to exclude those with significant cloud coverage, sun glint, and impacted by thick aerosols, 325 scenes were finally selected to track the long-term variation of Chl-a at Poyang Lake, using a normalized green-red difference index (NGRDI) [36].

The NGRDI is based on remote sensing reflectance (*Rrs*) at MERIS 560-nm and 681-nm bands, which relate to the surface Chl-a concentrations in Poyang Lake. The index is defined as: NGRDI = (*Rrs,_560_* − *Rrs,_681_*)/(*Rrs,_560_* + *Rrs,_681_*). To establish and validate the relationship between Chl-a and NGRDI, two field surveys were conducted; one in October 2009 (17–26 October), and the other in July 2011 (15–23 July), representing both the dry and wet conditions of Poyang Lake, respectively. There were 98 in situ data measurements that were collected in total. During these surveys, water samples were collected to determine Chl-a levels, together with surface reflectance values when weather conditions permitted. Detailed information about the in situ data used was derived from Feng, et al. (2015) [45]. An empirical exponential algorithm was then established that correlated the NGRDI and the Chl-a levels. It performs best among all possible linear, polynomial, power, or any other regression forms, with coefficients of determination (*R*^2^) of 0.7–0.8 and model uncertainties (e.g., MRE and RMSE) of <20%–40%.

The K-mean clustering method was then applied for spatio-temporal classification of the remote sensing Chl-a images from 2003 to 2012, which classifies pixels with similar temporal variation patterns into the same spatial cluster. Clustering is a process of grouping sets of objects based on their similarity, so that one object is more similar to another in the same cluster, and less similar to another in a different cluster, according to a given distance function. Clustering of time series data in this study aims to produce clusters with highly similar intra-cluster spatio-temporal variations, and low inter-cluster similarity. More specifically, pixels belonging to the same cluster exhibit high similarity to each other, and thus could be effectively represented using a typical observation and a lower pixel resolution, such as in the case of the in situ stations selected for this study.

### 3.2. Temporal Variation Analyses

**Figure 3:**
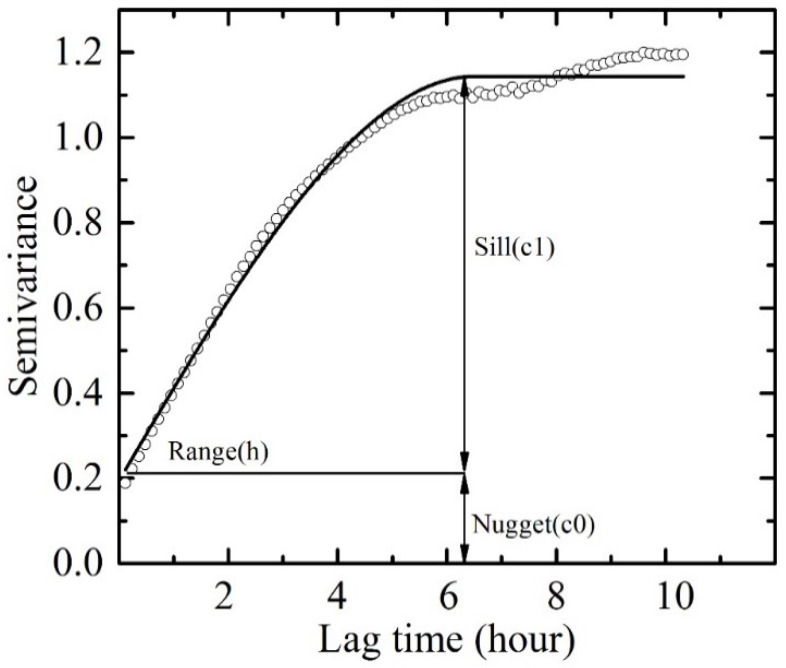
A spherical semivariogram showing range (*a*), sill (*c*) and nugget (*c*_0_). The dots represent experimental data and the solid line represents the model fit.

To quantify the temporal variation of Chl-a, a temporal scale analysis method was adopted. The temporal scale is defined as the decorrelation time of Chl-a time series data and can provide direct estimations of Chl-a variations. A semivariogram analysis is commonly used to characterize the spatial or temporal autocorrelation of variables separated by distance or time, and it indicates distance or time measurement separations beyond which the data become irrelevant [46,47,48]. The experimental semivariance (Equation (1)) was calculated as half the squared difference between measurements separated by different distances or times referred to as lag *h*:

(1) $$r(h)=\frac{1}{2N(h)}\sum_{j=1}^{N(h)}\left[z(x_{j})-z(x_{j}+h\right){]}^{2}$$

where z(*x_j_*) is the Chl-a concentration measured at time *x_j_*. *h* is the distance between data values, and *N(h)* are the number of pairs of data values a distance of *h* apart. By incrementing *h* in steps, we obtain an ordered set of values, as showed in Figure 3. To describe the temporal correlation structure objectively, the theoretical semivariogram (Equation (2)) is estimated from experimental semivariance using an ordinary least squares fit method (OLS).

(2) $$r^{\prime }(h)=c_{0}+c_{1}[(\frac{3h}{2a})-0.5(\frac{h}{a}{)}^{3}]$$

The OLS fit method minimises the error of the fitted theoretical model between the experimental data points (Equation (3)).

(3) $$min(error)=\sum \left(r(h\right)-r^{\prime }(h){)}^{2}$$

The theoretical semivariogram allows quantitative analysis of temporal variability based on three parameters: range (*a*), sill (*c*_1_), and nugget (*c*_0_), as illustrated in Figure 3. The sill (*c*_1_) represents the maximum variance of the dataset, and the nugget (*c*_0_) indicates the variation that remains unresolved including any measurement error. The range (*a*) determines the temporal scale over which the variance reaches its highest value and the observation becomes independent. In this study, the spherical model, one of the most commonly used theoretical semivariance models, was adopted to analyse the temporal scale of the Chl-a variation.

### 3.3. Sampling Error Analysis

A statistical method was employed to determine the optimal sampling strategy by analyzing the sampling error for different sampling times and intervals. High frequency Chl-a data were averaged for each half hour to create 48 measurements for each day at 30 min intervals (0:00, 0:30, 1:00, …, 23:00, 23:30). Three statistical indicators—the mean, maximum, and minimum values—were adopted for sampling error analysis.

The admissible sampling period for the satellite ocean color sensor data was first restricted to between 09:00 and 16:00 each day, to ensure stable light conditions and avoid significant surface directional reflectance or sun glint [49]. Then, the sampling frequency (i.e., temporal resolution) was estimated based on the designed satellite observation frequencies at 1/8, 1/4, 1/2, 1, and 2 days. For each scenario, the “satellite-measurement” daily mean, maximum, and minimum values were estimated using measurements taken at one specific moment in time, and these were compared with the “ground-truth” values estimated from the in situ high frequency measurements. The “ground-truth” daily mean, maximum, and minimum values were calculated from all of the available (48 measurements at 30 min interval) in situ data collected for the day. For instance, for the sampling frequency of 1/8, i.e., 8 measurements per day between 09:00 and 16:00 at 1-h intervals, the mean, maximum and minimum values of Chl-a were calculated. Finally, for a once-a-day scenario, the acquisition time was further set to between 09:00 and 16:00 at 30-min intervals to determine the optimal time for Chl-a measurements.

To assess the precision of the varied sampling strategy, the root mean square errors (Equation (4): *RMSE*) and mean differences (Equation (5): *bias*) were calculated as measures to describe the similarity/difference to the “ground-truth” data (Equation (6)). The RMSE and bias also indicate the presence of errors associated with insufficient measurements from the conventional satellites and provide a basis for future mission planning.

(4) $$RMSE=\sqrt{\frac{1}{N}\sum_{i=1}^{i=n}\left(x_{i}-y_{i}\right)}$$

(5) $$bias=(\frac{1}{n}\sum_{i=1}^{i=n}\varepsilon_{i})\times 100$$

(6) $$\varepsilon_{i}=\frac{x_{i}-y_{i}}{y_{i}}$$

where *x_i_* represents the Chl-a values observed using the sampling strategy, *y_i_* represents the in situ data, and n is the number of matching pairs. The observation frequency that produces the smallest *RMSE* and *bias* values therefore yields more objective estimations. A similar approach was applied to further determine the optimal time of day for observations of water quality that should be used for future satellite missions.

## 4. Results

### 4.1. Selection of the In-Situ Measurement Sites

**Figure 4:**
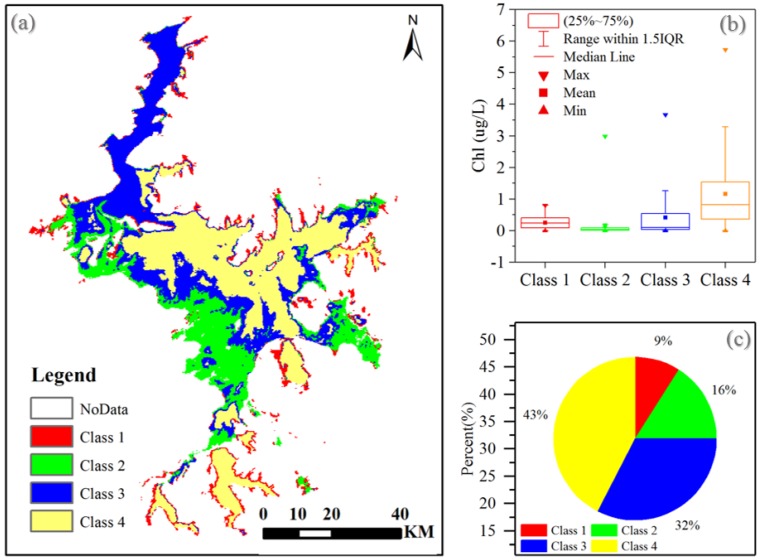
Classification of time series (2003–2012) MERIS derived Chl-a images: (**a**) 4 classes obtained from K-means cluster; (**b**) Chl-a range of each class; (**c**) area percentage of each class.

**Figure 5:**
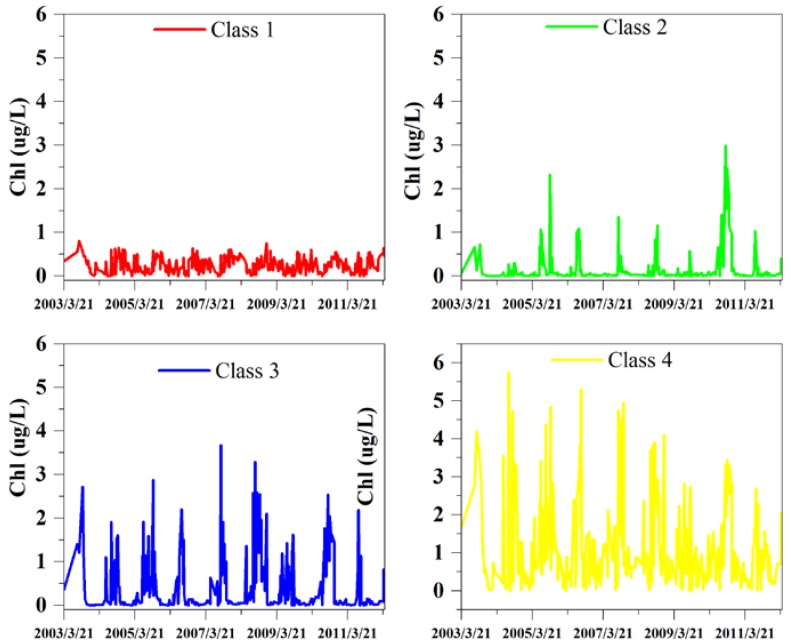
Temporal variations of Chl-a concentrations for the four classification regions.

The long-term Chl-a distribution showed significant spatial gradient and temporal variability, with Chl-a ranging from 2.4 ± 0.2 mg/m^3^ in April and 4.4 ± 1.0 mg/m^3^ in July, and no significant increasing or decreasing trend during the 10-year period. Figure 4 and Figure 5 present the results of K-mean lake surface class clustering based on 10 years of Chl-a images. 4 classes were obtained with distinct spatio-temporal patterns. The mean values and standard deviations show an increasing trend from class 1 to class 4, from 0.26 ± 0.18 mg/m^3^ for the first cluster up to 1.16 ± 1.12 mg/m^3^ for the last cluster. Results indicated that class 1 and class 2 had slight variation of Chl-a from 2003 to 2012, both spatially and temporally, while class 3 and class 4 went through larger variation. Moreover, 75% of the lake area was dominated by class 3 and class 4.

The field stations were, therefore, located in class 3 and class 4 areas to represent the most typical conditions of the lake. Site A was located at the north of the lake, where the mean Chl-a concentration was 1.8 µg/L, and the standard deviation was a small 0.9, while site B was located in an area with a higher Chl-a concentration (3.2 µg/L) and larger standard deviation (12.7). As a result, the field measurements and results from these two sites effectively represent the overall condition of Poyang Lake.

**Figure 6:**
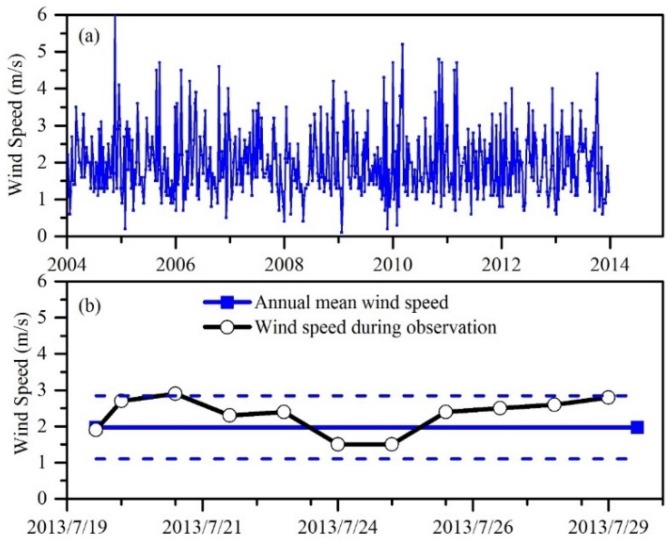
Wind speed at Poyang Lake: (**a**) daily time series results from the meteorological site, (**b**) comparison between annual mean wind speed and wind speed during field measurements.

Figure 6 shows multi-year wind speed data obtained from the Chinese Meteorological Data Sharing Service System. It showed that the daily mean wind speed during the observation period was close to the average wind speed for Poyang Lake. The data collected from these field sites was therefore obtained during a period of normal weather conditions, and so the field data could be used to represent the average state of the lake during in situ data collection, and for further analysis of short-term variations in water quality.

### 4.2. Validation of the Unattended Sensors Measured Chl-a

The comparison of lab fluorometric measured Chl-a and in situ ECO measured Chl-a levels was performed using 63 pairs of matchups collected during the daytime from in situ sensor locations. Statistics obtained, including the mean, standard deviation (STD), coefficient of variation (CV), minimum (Min), and maximum (Max) for Chl-a, are presented in Table 1. This indicated some variations between the two methods for Chl-a measurements. ECO Chl-a values were larger than lab fluorometric Chl-a values overall, and ECO measurements varied by around 25.33%, whereas lab fluorometric Chl-a values varied by 37.35% relative to the mean.

**Figure 7:**
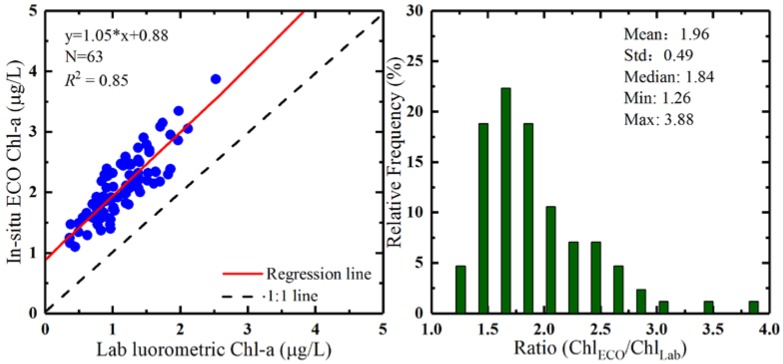
Regression analysis and comparison of lab fluorometric and ECO Chl-a.

A linear relationship between the lab fluorometric values for total Chl-a and the ECO fluorescence-derived values for Chl-a was obtained using regression analysis, as shown in Figure 7. The regression slope was fitted to be 1.05 with a correlation coefficient (*R*^2^) of 0.85. 

Chlorophyll fluorescence is influenced by photosynthetic active radiation (PAR), the absorptive properties of the phytoplankton, the extent to which the emitted fluorescence is reabsorbed, and the fluorescence yield [50]. Overall, it has been demonstrated that chlorophyll concentrations can be effectively estimated from fluorescence [26,51,52], and previous studies proved that chlorophyll estimation modelling from fluorescence can be effective at a regional scale.

**Figure 8:**
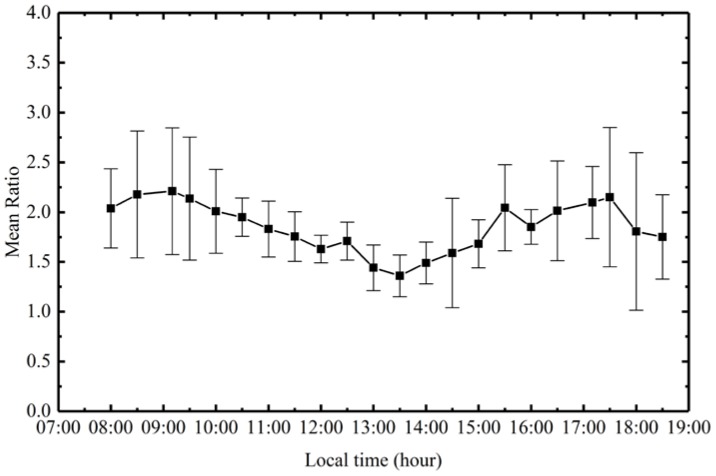
Diurnal variation of ECO Chl-a and lab fluorometric Chl-a ratio.

The ratio between in situ ECO measured Chl-a values and lab fluorometric measured Chl-a values ranged from 1.26 to 3.88, with a mean value of 1.96, as shown in Figure 7. The results from Poyang Lake were comparable to a regional analysis of the Arabian Sea coastal upwelling region, where a mean ratio of about 1 was observed. This value can vary significantly, for example a value greater than 6 was obtained in the Southern Ocean province south of New Zealand [26]. Figure 8 shows the diurnal variation of mean ratios observed during the in situ measurements (vertical bars represent standard deviation), between in situ ECO Chl-a values and lab fluorometric Chl-a values. The ratio of ECO Chl-a to lab fluorometric Chl-a values decreased from the morning and reached its lowest value at 13:00, and then increased before nightfall. Data for night hours were not available because no lab fluorometric Chl-a measurements were collected during the night time.

Similar diurnal variation patterns for chlorophyll fluorescence and/or ratios were observed in previous studies [53,54,55]. The mid-day depression of chlorophyll fluorescence was explained by the increase in quantum yield at the beginning and end of the “day”. This is part of the photo adaptive process, and the mid-day depression is probably related to photoadaptation of the reaction centres [50,56]. Fluorometers can more effectively retrieve phytoplankton chlorophyll-a concentrations in Case 2 waters and red tide areas. Although the chlorophyll fluorescence varies with the Chl-specific absorption rate and the variability in the fluorescence quantum yield [26], the fluorescence signal, when present, is uniquely attributable to chlorophyll-a. It is unlikely to be confused with anything else, such as yellow substance or suspended matter [56], and so it can be used to provide spatially and/or temporally restricted estimation models of chlorophyll-a concentrations.

### 4.3. Short-Term Variations of Chl-a at Poyang Lake

**Figure 9:**
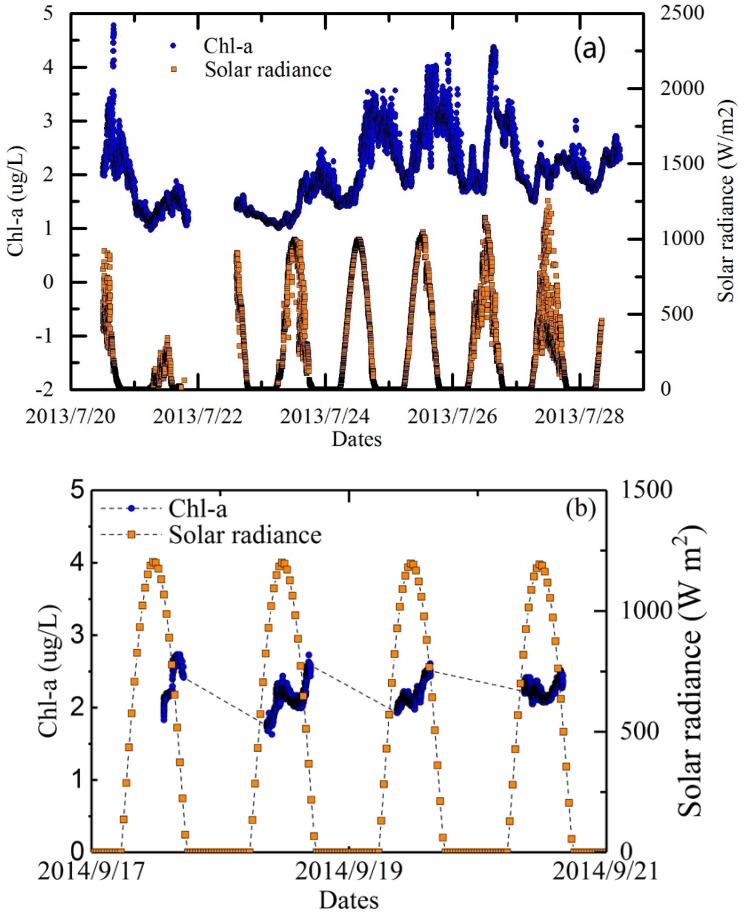
Short-term dynamics of Chl-a levels at site A (**a**) and site B (**b**) at Poyang Lake, together with solar radiance data collected simultaneously.

The results of the time series measurements demonstrated significant diurnal and inter-diurnal variations of Chl-a levels in Poyang Lake. Data collected from site A (2013) and site B (2014) are presented in Figure 9, respectively. The measurements were only performed during daytime; from 08:00 to 17:00 at site B. The break in the data obtained from site A, from 19:00 on 21 July to 14:00 on 22 July, was due to instrument maintenance.

Clear temporal variations in Chl-a (μg/L) levels were revealed by the high frequency field measurements at both sites A and B. Chl-a concentrations exhibited distinct daily periodic characteristics, with aspects of the fluctuations corresponding to the periodic changes in solar radiance, as shown in Figure 9. The Chl-a concentration was highest at approximately 15:00 during daytime, and lowest at night and in the early morning. An approximate 3 h time lag existed between the peak Chl-a values and peak solar radiance values, and the time lag was affected by non-photochemical quenching effects. Although characterized as a sediment-dominated lake [57], variations in Chl-a levels in Poyang lake were apparent. Table 2 shows that the temporal dynamic range of Chl-a was approximately 1 to 5 μg/L for both sites A and B.

The intra- and inter-diurnal variations of Chl-a levels in Poyang Lake were further investigated on a daily basis using time series measurements. 

**Figure 10:**
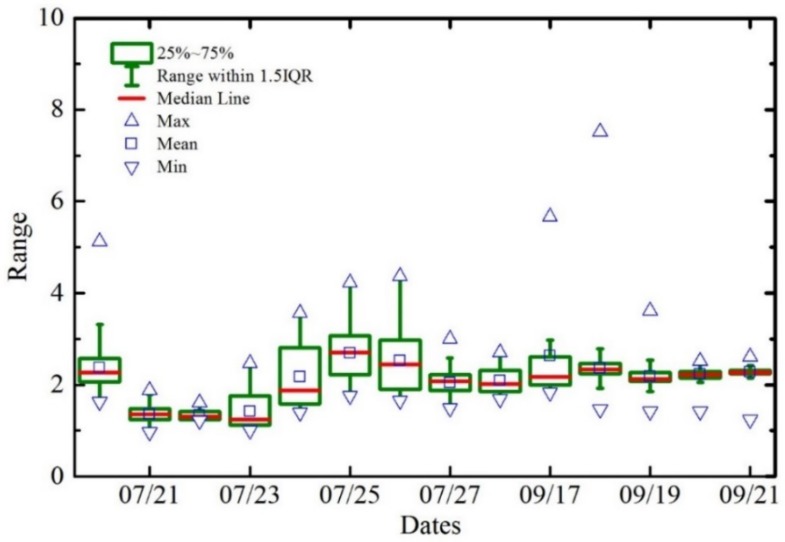
Boxplots representing the intra- and inter-diurnal variations of Chl-a at site A and B.

Figure 10 presents the statistical results from both site A and site B, including the full range of variation (from minimum to maximum), the interquartile range of variation (IQR), the mean, and the typical value (the median) of the daily Chl-a levels. The maximum diurnal ratio for Chl-a was 10.1, whereas the maximum inter-diurnal ratio was 34.8.

The results from the high-frequency measurements demonstrate the underlying variations of water quality, and revealed more rapid changes than conventional methods performed at monthly or seasonal frequencies could resolve. This shows that approaches with higher spatial and temporal coverage are more effective and should be used preferentially. Fortunately, such approaches are supported by advanced satellite water quality monitoring systems.

### 4.4. Temporal Scale of Chl-a Variations at Poyang Lake

**Figure 11:**
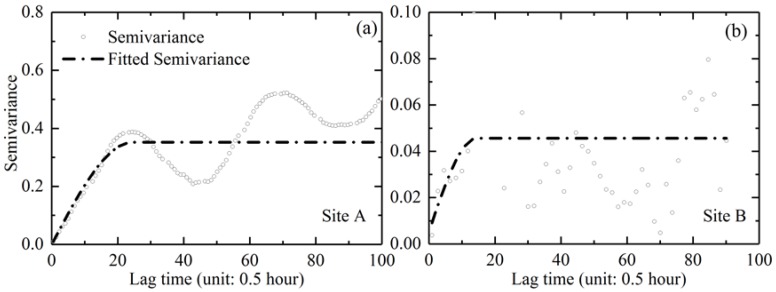
Temporal scales of Chl-a levels based on high-frequency measurements. The fitted spherical models are represented by the solid black lines.

The temporal scale determines the minimum time resolution needed to sufficiently resolve the dynamic water quality properties. Figure 11 shows the experimental and spherical model-fitted semivariance results of Chl-a levels at sites A and B, respectively, with a time lag of 30 min. Similar patterns were obtained with time lags of 1 h, 2 h, and 4 h, which are not presented here for brevity. However, statistical results for the temporal range, sill, and nugget for each site were obtained using the mean values from analyses of multiple time lags, as listed in Table 2.

All of the empirical semivariograms in Figure 11 showed certain distinct periodic patterns resulting from the internal daily cycles in the field measurement time series, despite certain fluctuations, as shown in Table 3. The oscillation periods were measured at approximately 1 day, as illustrated for both site A and B. The results again demonstrated significant diurnal variations of Chl-a levels at Poyang Lake, and the critical temporal scale was provided by the semivariogram analysis.

The theoretical variogram models were typically fitted through empirical semivariograms to quantify the temporal variability of Chl-a (*R*^2^ > 0.9, *P* < 0.01). Table 3 presents the mean values for range, sill, and nugget from the analysis of multiple lag times (1/2 h, 1 h, 2 h, and 4 h). The variogram sill (c1) is an indicator of the degree of temporal variability, and a larger sill (approximately 0.4) was found at site A. The nugget effect (c0) for the temporal variogram is significantly larger than zero at all lag times. This pattern implies the existence of certain unresolved smaller-scale temporal variability than even the 1/2 h time lag.

The temporal range (h) in the fitted variograms, which indicate suitable temporal resolutions to be utilized to capture the variations, is of great concern. For the Chl-a variation at sites A and B, the mean temporal range was determined to be approximately 12.6 and 6.6 h respectively, which is consistent with estimates from the semivariance plot. The results demonstrated the importance of higher frequency sampling of water quality monitoring involving Chl-a levels at the lake, especially in light of the recent elevated risk of eutrophication and cyanobacteria blooms.

Existing efforts dedicated to lake environment monitoring mainly rely on traditional ship-based field sampling techniques and space-based remote sensing. Despite the fact that more synoptic measurements obtained by remote sensing systems are used to complement the limited field observations, insufficient temporal coverage is still a primary barrier to more effective coastal and inland water monitoring. While more attention has been paid to higher temporal resolutions for coastal/inland remote sensing [13,49], our results highlight specific requirements that must be met in order to address the ever-increasing demands of water quality issues. A discussion of operational satellite sensors and future mission strategies is provided below.

## 5. Discussion

### 5.1. Temporal Gap Between Water Quality Variations and Existing Observation Approach

An estimation of Chl-a levels is typically one of the main objectives of water quality survey programs. Unbiased monitoring of Chl-a over large spatio-temporal scales requires well-designed monitoring strategies due to the highly dynamic properties of the water quality. Many factors may influence the accuracy of water quality monitoring over temporal and spatial scales, including radiometric calibration, atmospheric correction, and retrieval mode [58]. This study showed that sampling time and frequency can greatly influence the estimates of water quality factors, including Chl-a levels, in high-dynamic inland lakes, and that the chosen sampling strategy, including sample timing and frequency, can result in different estimates of water quality being obtained. This highlights the importance of adequate sampling frequency and appropriate sample timing during water quality surveys.

Existing efforts to monitor lakes in this context mainly rely on ship-based field sampling by local authorities, or remote sensing approaches developed by the wider scientific community. Most field surveys are conducted on a monthly/seasonal basis and some surveys may only be conducted at certain times of the year. For example, water quality survey is only conducted once a month at Poyang Lake by the Jiangxi Provincial Hydrographic Bureau [59], and monthly samples were also collected for three English lakes [60]. Environmental data were collected monthly from 178 lake surveys in Britain, between April and October each year, under the European Union Water Framework Directive (WFD) [61]. This archived survey data provides basic information for local authorities with respect to water quality monitoring. In order to better understand the dynamics of water quality, however, higher sampling frequencies need to be employed in order to capture the spatio-temporal dynamics of the water quality parameters under investigation.

Despite the more synoptic measurements produced by remote sensing systems, which are used to complement the limited field observations, insufficient temporal coverage is a primary obstacle to effective coastal and inland water monitoring. Conventional ocean color sensors are almost all polar-orbiting, including the CZCS, SeaWiFS, Terra/Aqua MODIS, and MERIS systems. Theoretically, these polar orbit satellite sensors provide comprehensive information of water properties at temporal scales of days to weeks [14,15]. This study has demonstrated that given the rapid temporal dynamics of the properties being measured, and frequent cloud coverage over coastal and inland water bodies, the ability of existing sensors to monitor highly dynamic waters is limited, especially for regions with significant diurnal or semi-diurnal variations [16,17,18]. This discrepancy between water quality variations and existing observational approaches is common and has also been observed for the East China Sea, Tampa Bay, the southern North Sea, Hangzhou Bay, and Taihu Lake [20,21], where significant diurnal dynamics of water quality occurred [22,23]. Caution should therefore be taken, and surveys should include both field sampling and remote sensing to estimate water quality in those high dynamics waters.

### 5.2. Chl-a Bias of Terra/Aqua MODIS from In-Situ Simulated Data

Current ocean color satellites, including Terra/Aqua MODIS, and NPP VIIRS, provide daily measurements for coastal/inland waters. Several GEO ocean-color instruments are available or are planned for launch in the coming decade, including GEO-CAPE (NASA) and GOCI-II (South Korea). These satellites are designed to have 0.5 h to 1 h revisit capacity. Evaluation of the ability of these existing and planned satellite missions is now crucial to determine if they can effectively sample the dynamics of coastal/inland waters. In this study, the uncertainties produced by satellite-derived Chl-a values were evaluated. The “satellite-measurement” daily mean, maximum and minimum values were obtained from the instantaneous remote measurements, and were compared with the “ground-truth” values estimated from the in situ high-frequency measurements. The “ground-truth” daily mean, maximum, and minimum values were calculated from all of the available in situ data for the day (48 measurements at 30 min interval).

**Figure 12:**
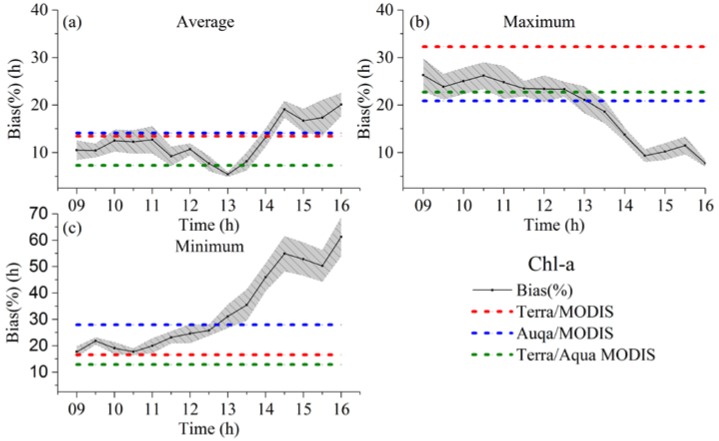
Biases (%) of the Chl-a level estimates for average (**a**), maximum (**b**), and minimum (**c**) as a function of measurement time

Figure 12 shows the biases (%) for the Chl-a level estimates (average, maximum, and minimum) as a function of measurement time. The time at which the biases are lowest was determined to be the optimal time for sampling. The biases of Terra/MODIS, Aqua/MODIS, and Terra/Aqua MODIS were also plotted for comparison.

The biases for the estimation of the average Chl-a concentration apparently decrease through the morning, and reach a trough at about 13:00. They then increased during the afternoon. The highest bias of 25% was observed at approximately 16:00 and the lowest bias of 5% was measured at approximately 13:00 (Figure 12). The Chl-a measurements from the Terra/MODIS or Aqua/MODIS were found to have a bias level ranging from 12% to 15%, implying that existing data products from a single satellite are potentially biased. However, seamless merging of Terra and Aqua MODIS data produces a bias level of less than 10%, although an inopportune observation could directly produce a bias greater than 30% and 50% for the maximum and minimum Chl-a estimations, respectively (Figure 12b,c). The optimal times for measuring were determined to be approximately 10:00 and 15:30 for the maximum and minimum Chl-a estimations, respectively. Similarly, the combination of Terra and Aqua MODIS data substantially improved the accuracy of the maximum and minimum Chl-a estimations.

### 5.3. Implications for Future Coastal/Inland Water Satellite Mission Plans

The bio-optical properties of coastal/inland waters are dominated by inter-diurnal or hourly variations that are optically complex and require more frequent sampling by satellite sensors than is currently available. This study revealed the importance of utilizing a temporal scale of less than half a day. Dedicated Chl-a monitoring efforts in Poyang Lake are still rare, despite the urgent need for this type of monitoring, given the increased eutrophication potential [34] and the high risk of cyanobacterial blooms in recent years (http://cnews.chinadaily.com.cn/2014-09/10/content_18576336.htm). Routine measurement of Chl-a levels are required to better understand the dynamics and driving forces of Chl-a under the combined influences of nutrient availability, freshwater flow input rates, and local human activity [42,43,62].

This scale of temporal resolution is also necessary to address the complex environments in other inland lakes and coastal zones. Using high-frequency monitoring, the short-term variability in the optical properties of Taihu Lake in China [29] and Tampa Bay in Florida (USA) [63] were effectively captured. Similarly, a minimum sampling frequency of three hours is required to fully resolve the effects of tides on salinity, turbidity, and Chl-a levels in coastal waters [64]. The feasibility of using geostationary satellite data for short-term temporal variability mapping of TSS concentrations in the southern North Sea has similarly been demonstrated [65].

**Figure 13:**
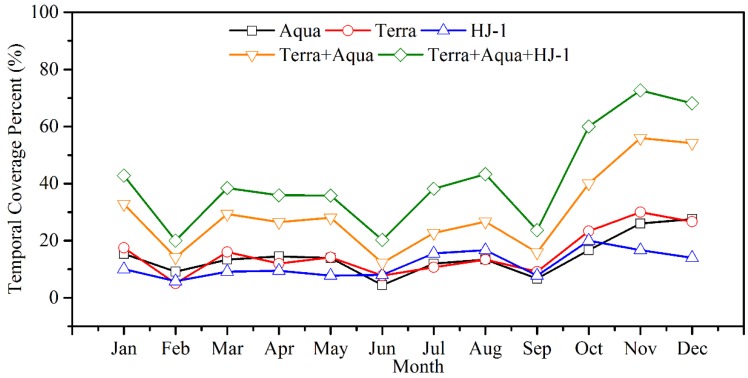
Temporal coverage in percent for different satellite sensors over Poyang Lake (data obtained from 2009 to 2016).

Ostensibly, the present temporal coverage by polar-orbiting satellites is one pass per day, which is not sufficient to capture the dynamics of the coastal/inland waters. Moreover, these regions are prone to cloud cover, which further limits the observation frequency from such satellites. The temporal coverage over Poyang Lake by the individual Terra/MODIS, Aqua/MODIS, and HJ-1 CCD sensors was less than 30% on average, especially during the rainy seasons, as shown in Figure 13. The coverage percent could be enhanced to approximately 50% by using data from multiple remote sensors, but the coverage still needs further improvement to achieve the goal of twice-a-day measurements.

Within this context, one promising solution lies in advances in geostationary remote sensing technology, following the encouraging progress of coastal/inland monitoring by the SEVIRI system, aboard the Meteosat Second Generation [24], and the South Korean Geostationary Ocean Color Imager (GOCI) [19,66]. Other space agencies around the world have expressed interest in geostationary ocean color remote sensing, including the high-resolution ESA GEO mission (Europe), the NASA geostationary mission (GEO-CAPE; USA), and the CNSA high-resolution geostationary GF-4 mission (China). The conclusions drawn from the current study, focused on the largest freshwater lake of China, highlight specific sample frequency and sample time requirements for the design of future GEO missions. Moreover, for the region in question, it would be most desirable if future satellite missions have afternoon overpass times, because most of the previous and operational ocean color missions feature polar orbits with morning overpass times. 

## 6. Conclusions

In this study, the short-term dynamics of Chl-a concentrations at Poyang Lake were addressed for the first time, with emphasis on optimizing sampling strategies for future water quality monitoring. Using the high-frequency stationary site measurements we were able to effectively characterize the intra- and inter-diurnal variability of Chl-a levels. The large variations in the lake were demonstrated with the maximum intra-diurnal ratio for Chl-a was 10.1, and the maximum inter-diurnal ratio was 34.8. The temporal dynamic range of Chl-a was determined to be 9.6 h on average, using semivariogram analysis. The analysis of optimal sampling strategies revealed that the effect of sampling time on mean Chl-a concentrations was higher than 25%, and the uncertainty of single Terra/MODIS or Aqua/MODIS observation was 15%. With respect to the fundamental design of remote sensing systems for monitoring coastal/inland waters, the study demonstrated that future satellite missions should aim for higher temporal frequencies (at least twice a day) and appropriate measurement times (increasing the proportion of afternoon overpasses). The results of this study highlight specific requirements that should be utilized for the design characteristics of the GEO missions currently in progress and in planning, which will help address the challenges of monitoring complex coastal and inland environments around the world.

## Figures and Tables

**Table 1:** Statistics and comparisons of HPLC-Chl and ECO-Chl.

Variables	Mean	STD	CV (%)	Min	Max
HPLC-Chl	1.15	0.43	37.35	0.36	2.53
ECO-Chl	2.09	0.53	25.33	1.10	3.87

**Table 2:** Statistics of Chl-a (μg/L) at of Poyang Lake in 2013 and 2014.

	Mean	Std	CV	Range	Number of Samples
Site A	2.04	0.35	0.17	0.97–4.92	50,701
Site B	2.17	0.23	0.12	1.71–2.83	2547

**Table 3:** Statistics of the temporal scale of Chl-a at Poyang Lake.

		Sill (*c*_1_)		Nugget (*c*_0_)		Range (*h*)	
		Mean	Std	Mean	Std	Mean	Std
Chl-a (μg/L)	Station A	0.40	0.09	0.06	0.09	12.56	1.49
	Station B	0.04	0.03	0.01	0.03	6.57	10.33
